# Evaluation of Haematological Ratios at: Different Stages of Canine Periodontal Disease

**DOI:** 10.3390/vetsci11110581

**Published:** 2024-11-19

**Authors:** Carolina Silva, Ana Carolina Abrantes, Ana Carolina Fontes, Isabel Dias, Rosário Domingues, Francisco Peixoto, Carlos Viegas

**Affiliations:** 1 Department of Veterinary Sciences, School of Agricultural and Veterinary Sciences (ECAV), University of Trás-os-Montes e Alto Douro (UTAD), Quinta de Prados, 5000-801 Vila Real, Portugal; al57891@alunos.utad.pt (C.S.); carolina.psca@gmail.com (A.C.A.); al61909@alunos.utad.pt (A.C.F.); idias@utad.pt (I.D.); 2 Animal and Veterinary Research Center (CECAV)—AL4AnimalS, University of Trás-os-Montes e Alto Douro (UTAD), 5000-801 Vila Real, Portugal; 3 CITAB-Centre for the Research and Technology of Agro-Environmental and Biological Sciences—Inov4Agro, University of Trás-os-Montes e Alto Douro (UTAD), 5000-801 Vila Real, Portugal; 4 Mass Spectrometry Centre, LAQV-REQUIMTE, Department of Chemistry, University of Aveiro, Campus Universitário de Santiago, 3810-193 Aveiro, Portugal; mrd@ua.pt; 5 CESAM, Departament of Chemistry, University of Aveiro, Campus Universitário de Santiago, 3810-193 Aveiro, Portugal; 6 Chemistry Center of Vila Real, University of Trás-os-Montes e Alto Douro (UTAD), Quinta de Prados, 5000-801 Vila Real, Portugal; fpeixoto@utad.pt; 7 RISE-Health: Health Research Network, Faculty of Medicine, University of Porto, 4099-002 Porto, Portugal; 8 CIVG—Vasco da Gama Research Center, University School Vasco da Gama (EUVG), Campus Universitário, Avenida José Rodrigues Sousa Fernandes, Lordemão, 3020-210 Coimbra, Portugal

**Keywords:** haematological ratios, dog, gingivitis, periodontal disease, periodontitis, Podengo Portuguese Bbreed, ratios, systemic inflammatory response

## Abstract

This is a retrospective study about haematological ratios in different stages of canine periodontal disease in one single breed, more specifically the Portuguese Podengo. The aim of the study was to assess the clinical significance of five haematological ratios, namely neutrophil-to-lymphocyte ratio (NLR), platelet-to-lymphocyte ratio (PLR), mean platelet volume-to-platelet count ratio (MPV/PLT), monocyte-to-lymphocyte ratio (MLR), and platelet-to-neutrophil ratio (PNR), easily obtained through the parameters provided by the complete blood count, in the stages of gingivitis and periodontitis, compared to clinical healthy individuals. Receiver operating characteristic curves were generated to identify ideal sensitivity and specificity cut-offs for cases of gingivitis or periodontitis. Our study included 80 dogs, which comprised 24 healthy dogs, 26 diagnosed with gingivitis, and 30 with periodontitis. The median NLR was significantly lower in periodontitis than in clinical healthy dogs (*p* = 0.040) and in dogs with gingivitis (*p* = 0.037). The median PLR was significantly lower in cases of gingivitis (*p* = 0.020) and periodontitis (*p* = 0.024) than in healthy dogs. MPV/PLT and MLR did not demonstrate significant differences between any of the three groups. The median PNR was significantly lower in gingivitis than in control dogs (*p* = 0.019). PNR had the highest accurate results, in distinguishing between healthy individuals and those with gingivitis, with an area under the curve of 0.692 (95% CI [0.539–0.845], *p* = 0.020). However, even this index had weak discriminatory power between the groups in the analysis. The impact of systemic inflammation generated by canine periodontal disease can thus be seen through some of these indices. However, the results obtained here demonstrate the need for complementarity with other methods to diagnose canine periodontal disease and reinforce the need for additional studies with more markers of systemic inflammatory response.

## Simple Summary

Periodontal disease is a highly prevalent disease in the canine population and has a considerable systemic impact. Blood count indices have been investigated in many different diseases but, so far, no studies have been carried out on their value in different degrees of canine periodontal disease. The aim of the present study was to investigate and analyse five different haematological ratios in healthy clinical dogs, dogs with gingivitis, and dogs with periodontitis and, secondarily, evaluate their potential as diagnostic biomarkers. This retrospective study included 80 dogs of a specific breed and with different stages of canine periodontal disease. We found statistically significant differences between the groups in three of the haematological ratios analysed. These results suggest the systemic impact that canine periodontal disease has and reinforces the need of further research in this area.

## 1. Introduction

Canine periodontal disease (PD) is one of the most prevalent conditions diagnosed in small animal clinic [1,2]. Its prevalence is so high that some studies show that 80% of dogs around two years old already have PD [3]. However, the disease is more common in older dogs [4] and the most frequently affected breeds are the small and toy breeds [5,6].

The susceptibility and clinical expression of PD are conditioned by its multifactorial aetiology, which includes behavioural, environmental, genetic, microbiological, and systemic factors [7].

PD is a progressive infectious-inflammatory disease characterised by two different stages: gingivitis and periodontitis, where destruction of the periodontium tissue is induced by the immune response generated by the individual, due to the bacterial biofilm on the tooth surface, and by the direct action of this same bacterial biofilm [2,5,8,9,10]. Gingivitis is considered the first stage of PD, in which inflammation can be seen along the gingival margin and haemorrhage can occur. However, there is no loss of connective tissue or alveolar bone attachment. In some cases, gingivitis can develop into periodontitis if it is not treated early and properly. Periodontitis, on the other hand, is an irreversible and painful condition. It is characterised by a chronic inflammation and destruction of the supporting tooth tissues, namely the gingiva, the alveolar bone, the periodontal ligament, and the cementum [3,11,12]. As the disease progresses, it can lead to tooth mobility, tooth loss, periodontal abscesses, oronasal fistulas, or even osteomyelitis of the maxilla and mandible [3,11,12].

A dog’s general health may be significantly impacted by PD, even in its early stages [13,14]. An article by Whyte et al. [13] demonstrated this. The degree of dental plaque in dogs diagnosed with PD at an early stage, specifically stage 1, was correlated with alterations at platelet level and at liver level, through alanine aminotransferase [13]. This shows that even at an early stage, the impact of PD is already significant [13]. It is known that there is a systemic inflammatory response that is generated in PD patients, whether they are humans or dogs [15,16]. In the case of canine PD, it has been linked to endocardiosis and endocarditis, hepatitis, interstitial nephritis, chronic bronchitis, and pulmonary fibrosis [17,18]. These comorbidities are boosted by the dissemination of bacterial metabolic products from periodontopathogens into the systemic circulation, which triggers the host’s responses [15,16]. In turn, this systemic inflammatory response has been associated with an increase in systemic inflammatory mediators in dogs and humans [19,20,21,22]. Consequently, PD must be seen as a disease that can seriously jeopardise the individual’s life. Veterinarians should express to the owners the real impact of this disease, which is certainly not a localised dental pathology [8].

Complete blood count (CBC) tests are routinely used in clinical small animal practice. Using the CBC, it is possible to easily calculate various ratios, including the neutrophil-to-lymphocyte ratio (NLR), platelet-to-lymphocyte ratio (PLR), mean platelet volume-to-platelet count ratio (MPV/PLT), monocyte-to-lymphocyte ratio (MLR), and platelet-to-neutrophil ratio (PNR), which can be used as important systemic inflammatory indicators. When compared to individual inflammatory cells, these haematological ratios (HRs) provide a more comprehensive evaluation of the inflammatory response. This is achieved because the interactions between various types of cells are taken into account and leads to greater precision in determining the immunological and inflammatory state [23,24]. In light of this, these indices are good indicators of systemic inflammatory responses and have the potential to be used as biomarkers for diagnosis and prognosis in cardiovascular diseases and inflammatory and neoplastic conditions [25,26].

Neutrophils are fundamental elements of the innate immune system and participate in the body’s defence against pathogens, as they are the first circulating phagocytic cells [27]. Their process of phagocytising potential pathogens takes place due to the intervention of inflammatory mediators, which stimulate the migration of neutrophils into the blood [27]. In turn, lymphocytes also deserve to be highlighted for their role in specific anti-inflammatory and antitumour immunity [16,28]. Monocytes, the largest group of white blood cells, are immature cells that are either freely moving through the bloodstream or on their way to a specific tissue [29]. Mature macrophages are created when a young monocyte enters tissue and leaves the bloodstream [29]. Due to the complex interaction between monocytes and macrophages with blood and tissues, they are referred to as a single system, or the mononuclear phagocyte system [29].

Leukocyte ratios, particularly the PLR and the NLR, have been the subject of much research in human medicine as biomarkers of inflammation and inflammation-related diseases [30]. These ratios have shown potential as prognostic indicators in patients with COVID-19, cancer, cardiovascular disease, pneumonia, and sepsis [30,31,32,33,34]. However, in contrast to human medicine, the NLR is not a reliable indicator of the prognosis for dogs with pneumonia and has no relation to the duration of hospitalisation or mortality in dogs with septic peritonitis [35,36]. NLR in veterinary medicine has already been studied in pathologies such as lymphomas [37], mast cell tumours [38], and sarcomas [39]. In dogs and cats with acute pancreatitis and acute diarrhoea, NLR is a helpful indicator of the prognosis and severity of the condition [40]. Moreover, the use of NLR makes it possible to distinguish dogs with meningoencephalitis of unknown origin from other forebrain disorders [41].

Platelets are another blood element worth mentioning. They are fundamental for homeostasis but also play a significant role in the inflammatory process [29,42,43]. They actively participate in the induction of inflammation and tissue repair, as well as coregulate the host’s defence against microbes [29,42,43]. Thrombocytosis is associated with various inflammatory, metabolic, and neoplastic processes [44]. In human medicine, thrombocytosis is even used as a prognostic marker in many neoplastic diseases [45]. PLR is calculated as a ratio between platelet and lymphocyte counts. These biomarkers reflect the balance between immunomodulatory and inflammatory processes (as reflected by the platelet count) and immunity response (lymphocyte count) [30]. In addition, PLR has been the subject of research into various canine pathologies [40,46,47], namely in canine parvovirosis, where it may even have prognostic value [30]. It has been shown that in dogs with serious haemorrhage, the PLR correlates with the period of hospitalisation in the intensive care unit. Moreover, the PLR and NLR correlate with the severity of the disease [30]. PLR and NLR are also inflammatory markers in canine inflammatory bowel disease and may be helpful in estimating the result of treatment [48,49,50]. Regarding platelets, another parameter is the mean platelet volume (MPV), which has also been the extensively studied, particularly in the field of oncology [51,52]. However, its role has yet to be clarified, so combining it with the total platelet count is recommended [16].

In the specific case of MLR, there are studies that prove that its increase, together with NLR, is associated with human cardiovascular diseases such as adverse cardiac events, mitral valve disease, coronary artery disease, and stroke [53,54]. However, in veterinary medicine, too, these HRs have been shown to have potential as diagnostic and prognostic biomarkers in myxomatous mitral valve disease in dogs [26].

Finally, the PNR resulting from the ratio obtained between platelets and neutrophils still needs to be investigated. However, there is already a study on this in the field of oncology, more specifically in canine diffuse large B-cell lymphoma [55].

Although HRs are being used more and more routinely in human medicine and in a wide variety of diseases, there is still a long way to go in veterinary medicine to demonstrate the potential value of these indices. So far, we know that blood count indices such as NLR, MLR, and PLR have shown potential as biomarkers of the systemic inflammatory response, such as in canine pancreatitis [40], neoplastic diseases [16,38], or sepsis [46]. However, no study has yet been carried out comparing certain blood count indices in different stages of canine PD, and even less so in a single breed. The aim of this study was to determine the clinical significance and compare the NLR, PLR, MPV/PLT, MLR, and PNR in dogs diagnosed with different degrees of canine PD. The authors of this study hypothesised that these HRs could be affected by the stage of canine PD and consequently be considered as potential biomarkers for distinguishing the different stages.

## 2. Materials and Methods

### 2.1. Data Collection and Analysis

The study is a retrospective case–control study. We chose to select just one breed for this study, the Portuguese Podengo, more specifically the medium-sized breed. The clinical records of 80 Portuguese Podengo dogs belonging to different owners were reviewed and analysed retrospectively from May 2020 to June 2024. The owners of the animals included in the study sample were asked to consent to the use of their animals’ data for research purposes.

Blood samples were previously obtained by puncturing the jugular veins, collected in tubes with EDTA anticoagulant, and processed immediately. It should be mentioned that all the blood samples were taken during the daytime in order to avoid any possible influence of the circadian rhythm on the parameters being analysed. The analyses were carried out on an automatic analyser (BC-5000 Vet, Mindray, Fujifilm Portugal, S.A., Vila Nova de Gaia, Portugal) and a complete study of the leukocyte, erythrocyte, and platelet series was obtained. Blood analyses were carried out in cases of medical prophylaxis based on an appointment with the assistant veterinarian.

### 2.2. Inclusion Criteria

The animals were divided into three different groups according to their PD condition, i.e., healthy, gingivitis, or periodontitis. All the dogs in the three groups had to present an unaltered general clinical examination, be properly vaccinated and dewormed, and not present any obvious analytical alterations compatible with any infectious, neoplastic, endocrine, renal, or hepatic pathology.

### 2.3. Exclusion Criteria

The exclusion factors were as follows: age under 1 year, presence of systemic pathology, being under medical treatment and females in heat, pregnant, or lactating. In addition to these factors, we also considered that all animals that had undergone surgery or periodontal treatment in the last 12 months would not be considered for the study sample.

### 2.4. The Characterisation of Each of the Three Groups Under Study

The group of healthy animals, known as the control group (C group), included 24 dogs. None of them showed even the slight presence of dental plaque or dental calculus. Nor could they show any evidence of gingival inflammation or alterations to the height or architecture of the alveolar margin.

The second group of dogs consisted of 26 dogs with clinical signs of gingivitis and was therefore called the gingivitis group (G group). The animals included in this group had superficial inflammation of the gingiva, with marginal erythema and oedema. They may also show bleeding on touch or slight spontaneous bleeding. It should be emphasised that the size and architecture of the alveolar margin are normal and there is no loss of insertion, although there may be a slight accumulation of dental calculus.

The third and final group in this study corresponded to dogs diagnosed with periodontitis (P group). This group included 30 dogs. These animals had to show signs of periodontitis, such as gingival recession, furcation exposure, tooth mobility, or even tooth loss. In addition to these signs, those mentioned above for G group also had to be present.

### 2.5. Calculation of Haematological Ratios

The HRs calculated were NLR, PLR, MPV/PLT, MLR, and PNR. The NLR was calculated as the ratio between the absolute value of neutrophils to lymphocytes. PLR and MLR were calculated as the ratio of the absolute values of platelets and monocytes to lymphocytes, respectively. The MPV/PLT was calculated as the ratio between the mean platelet volume and the absolute value of platelets. Finally, PNR was calculated as the ratio between the absolute value of platelets and neutrophils. In addition to these data, information was also collected on the age, gender, and weight of each animal. In particular, with regard to age and gender, statistical analyses were carried out on the groups in the study in order to ascertain whether age and gender influences the values of the HR under study.

### 2.6. Statistical Analysis

Statistical analyses were performed using Jamovi statistical software (version 2.3.28) and IBM SPSS statistics (version 29.0.0.0). The continuous variables were assessed for normality using the Shapiro–Wilk test. Normally distributed variables were reported as mean ± standard deviation. On the other hand, not normally distributed data were presented by the median and 25th and 75th percentiles. Laboratory variables were compared between the different groups using the Student’s *t*-test and the Mann–Whitney non-parametric test, depending on whether the variable was normally or not normally distributed, respectively. The influence of gender and age on the HRs studied in the three groups was analysed using the Mann–Whitney non-parametric test and simple linear regression, respectively. A receiver operating characteristic (ROC) curve analysis was used to identify cut-off levels of sensitivity and specificity for the HR that showed a statistically significant difference between the C group and one of the other two different groups. The area under the curve (AUC) was calculated and the cut-off values that optimised sensitivity and specificity were found using the Youden index. A *p*-value < 0.05 was considered statistically significant.

## 3. Results

### 3.1. Study Population

Table 1 summarises the main characteristics of the study sample (*n* = 80), comprising a total of 24 healthy dogs, 26 dogs with gingivitis, and 30 dogs with periodontitis.

All the parameters provided by the CBC and statistical analysis between the three groups are described in Table 2, where different letters represent statistically significant differences.

### 3.2. Haematological Ratios

Table 3 summarises the statistical results obtained by analysing gender and age in relation to the HRs in the C, G, and P groups.

The median of the five different HRs is shown in Table 4 for each of the three groups under study, covering a total of 80 individuals.

#### 3.2.1. Neutrophil-to-Lymphocyte Ratio (NLR)

**Figure 1:**
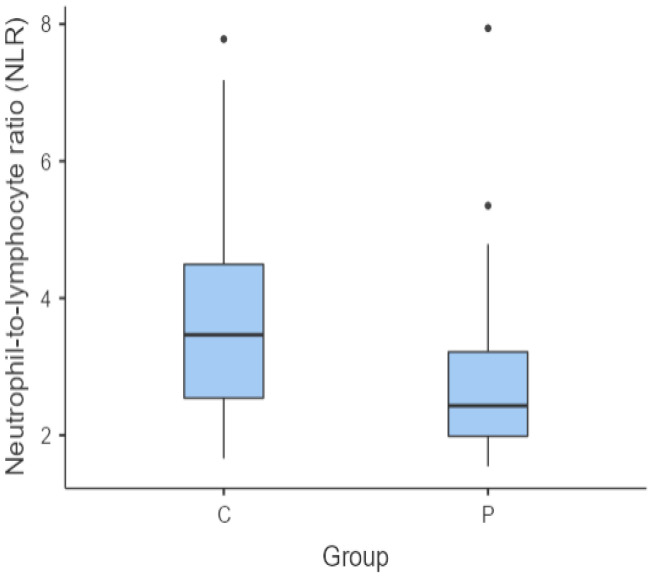
Box and whisker plot comparing the NLR for healthy individuals (C group) versus individuals diagnosed with periodontitis (P group). The central horizontal lines indicate the median value; the upper and lower lines of the box plot represent the first and the third quartiles of the values, respectively. The outliers are represented with circles.

**Figure 2:**
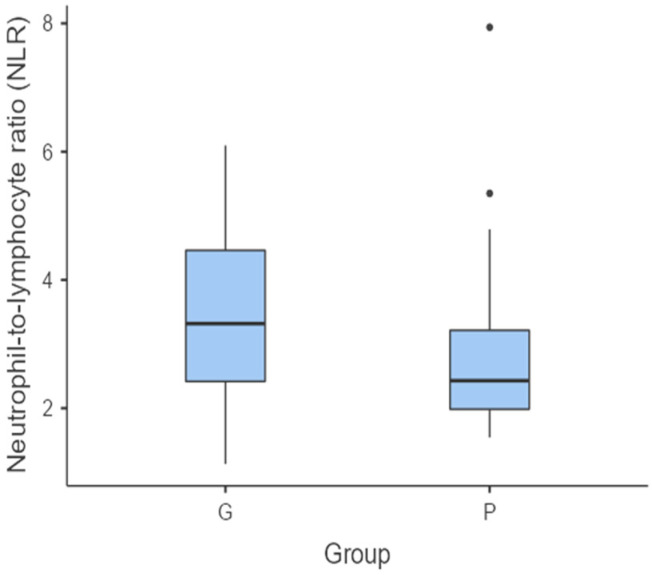
Box and whisker plot comparing the NLR for subjects diagnosed with gingivitis (G group) versus those with periodontitis (P group). The central horizontal lines indicate the median value; the upper and lower lines of the box plot represent the first and the third quartiles of the values, respectively. The outliers are represented with circles.

The NLR showed no statistically significant difference between the individuals in the C group and those in the G group (*p* = 0.751). However, this was not the case for the C group versus P group (*p* = 0.040) (Figure 1) and the G group versus P group (*p* = 0.037) (Figure 2). In these cases, there were statistically significant differences between the groups for the NLR, with the median of this haematological ratio being lower in the P group.

#### 3.2.2. Platelet-to-Lymphocyte Ratio (PLR)

**Figure 3:**
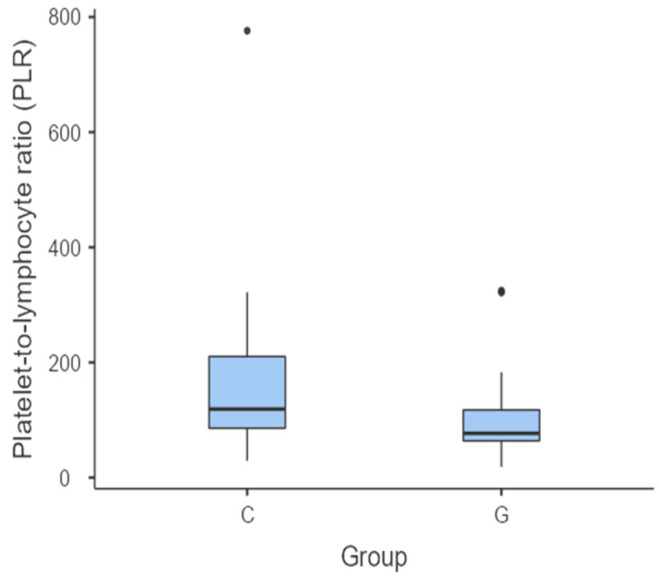
Box and whisker plot comparing the PLR between the C group and G group. The central horizontal lines indicate the median value; the upper and lower lines of the box plot represent the first and the third quartiles of the values, respectively. The outliers are represented with circles.

**Figure 4:**
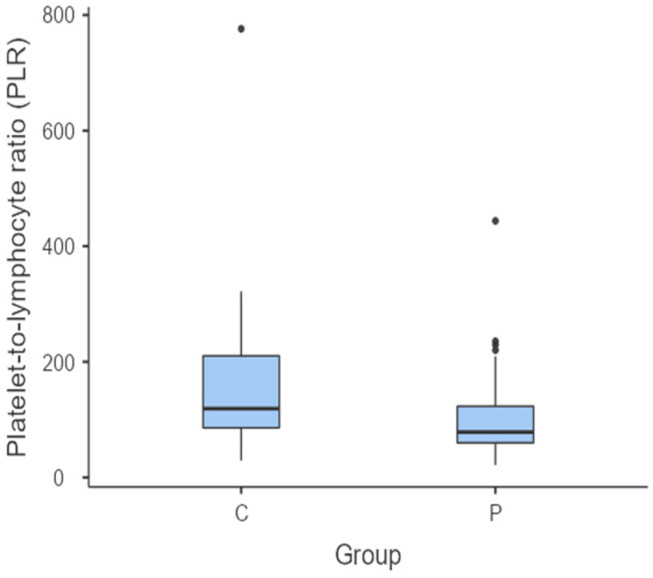
Box and whisker plot comparing the PLR between dogs considered clinically healthy (Group C) and dogs diagnosed with periodontitis (Group P). The central horizontal lines indicate the median value; the upper and lower lines of the box plot represent the first and the third quartiles of the values, respectively. The outliers are represented with circles.

The median PLR in the C group was the highest (119 (85.7–210)). The PLR was significantly higher between the C group and the G (*p* = 0.020) (Figure 3) and P groups (*p* = 0.024) (Figure 4). However, there were no statistically significant differences between the G and P groups in terms of the PLR (*p* = 0.864).

#### 3.2.3. Mean Platelet Volume-to-Platelet Count Ratio (MPV/PLT)

No statistically significant differences were found in any of the three MPV/PLT analyses carried out between the various groups.

#### 3.2.4. Monocyte-to-Lymphocyte Ratio (MLR)

The MLR did not differ significantly in the comparisons made between the different groups, i.e., between group C and group G (*p* = 0.293), group C and group P (*p* = 0.054), and between group G and group P (*p* = 0.217).

#### 3.2.5. Platelet-to-Neutrophil Ratio (PNR)

**Figure 5:**
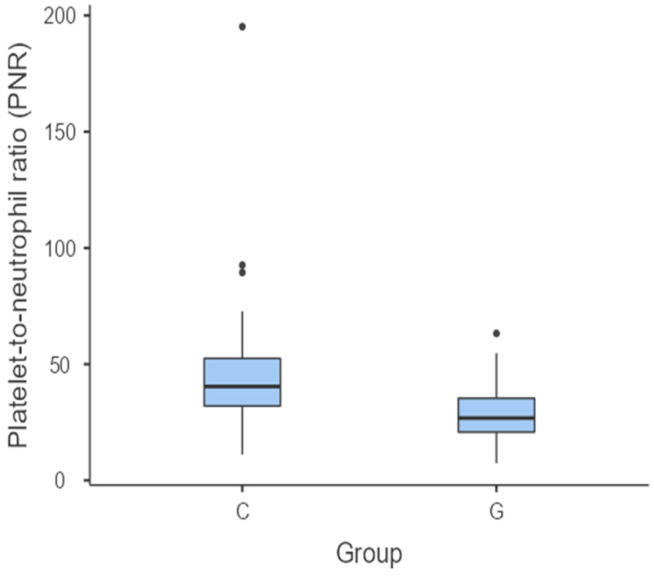
Box and whisker plot comparing the PNR between the C group versus P group. The central horizontal lines indicate the median value; the upper and lower lines of the box plot represent the first and the third quartiles of the values, respectively. The outliers are represented with circles.

The PNR was significantly higher in C group in comparison with G group (*p* = 0.019) (Figure 5). On the other hand, there were no statistically significant differences in the comparisons made between C group versus P group (*p* = 0.185) and G group versus P group (*p* = 0.166).

### 3.3. ROC Curves

**Figure 6:**
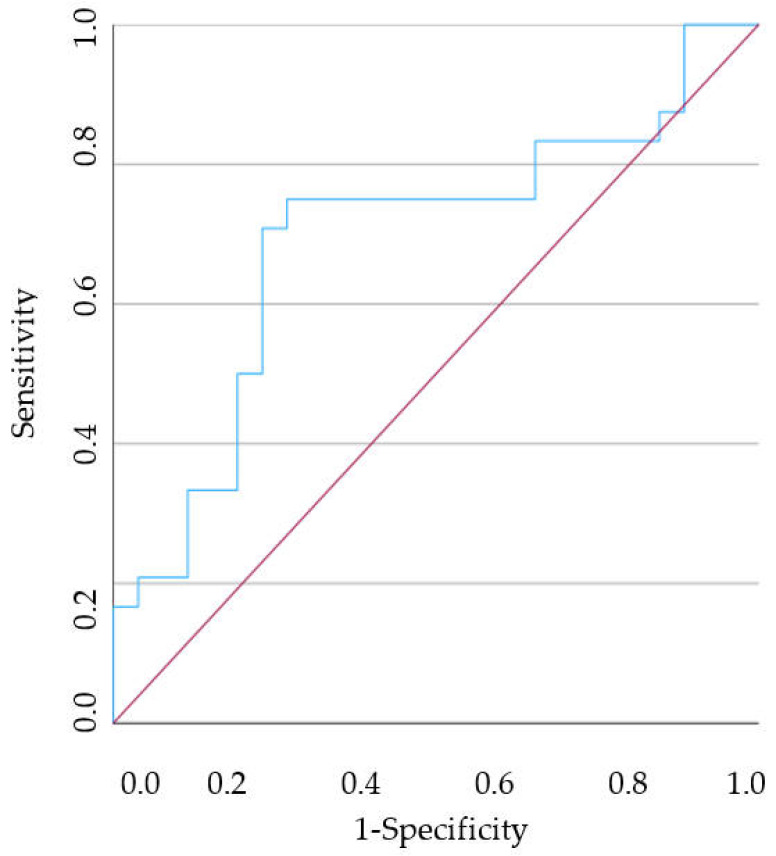
The ROC curve analysis of predicting the PNR between healthy dogs and dogs with gingivitis. The red line illustrates what a random classification would look like, and the blue line represents the model under study.

ROC curves were generated for the HR that demonstrated a statistically significant difference between healthy individuals and cases with gingivitis or periodontitis. It should be noted that the PNR showed the most accurate results, particularly in distinguishing between healthy individuals and those with gingivitis, with an AUC of 0.692 (95% CI [0.539–0.845], *p* = 0.020) (Figure 6). In this case, a cut-off of <34.490 demonstrated a 75.0% sensitivity and 73.1% specificity for individuals with gingivitis. On the other hand, the PLR was the only haematological ratio with statistically significant differences between the C group and the other two groups under study, which consequently led to the analysis of two different ROC curves. However, given the AUC values obtained for the HR analysed, the discriminating power of the models is considered weak, since all the AUCs are in the [0.5; 0.7] range [56].

**Figure 7:**
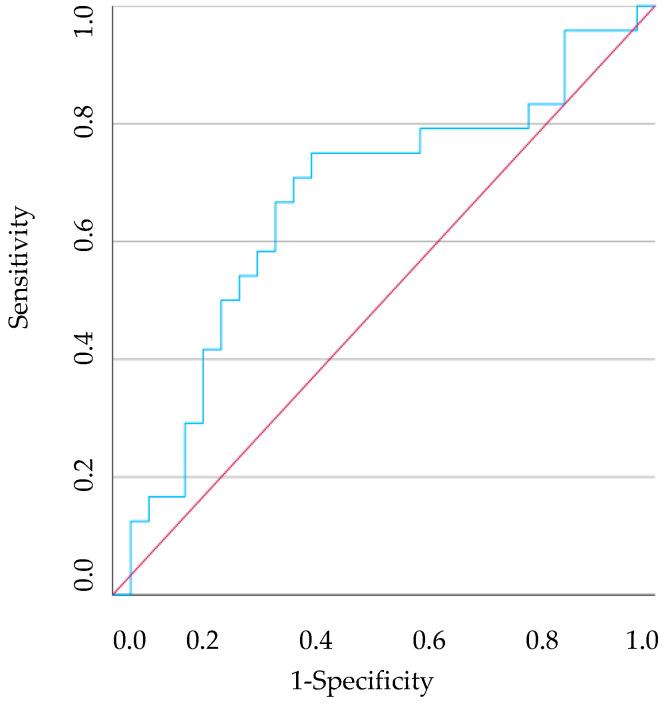
The ROC curve analysis of predicting the NLR between healthy dogs and dogs with periodontitis. The red line illustrates what a random classification would look like, and the blue line represents the model under study.

The AUC using the NLR was 0.0664 (95% CI [0.513–0.815], *p* = 0.040) (Figure 7). A cut-off of <2.577 showed a 75% sensitivity and 63.6% specificity for periodontitis cases.

**Figure 8:**
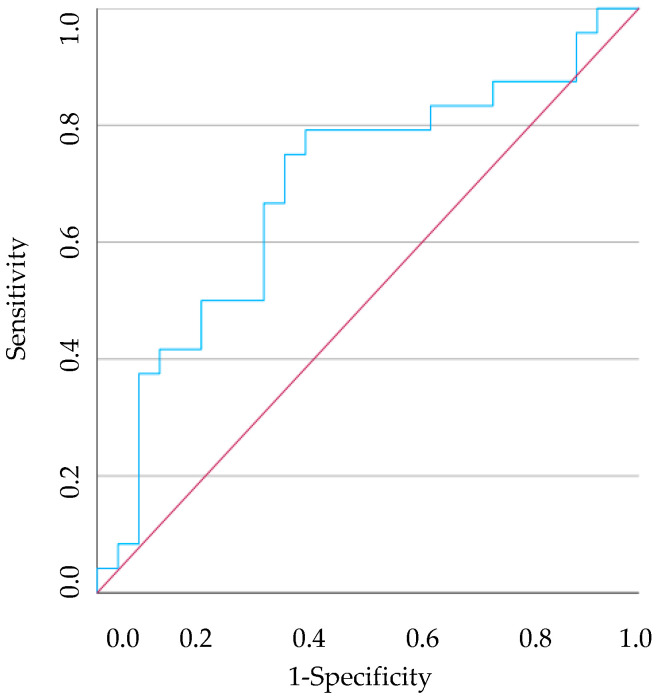
The ROC curve analysis of predicting the PLR between healthy dogs and dogs with gingivitis. The red line illustrates what a random classification would look like, and the blue line represents the model under study.

**Figure 9:**
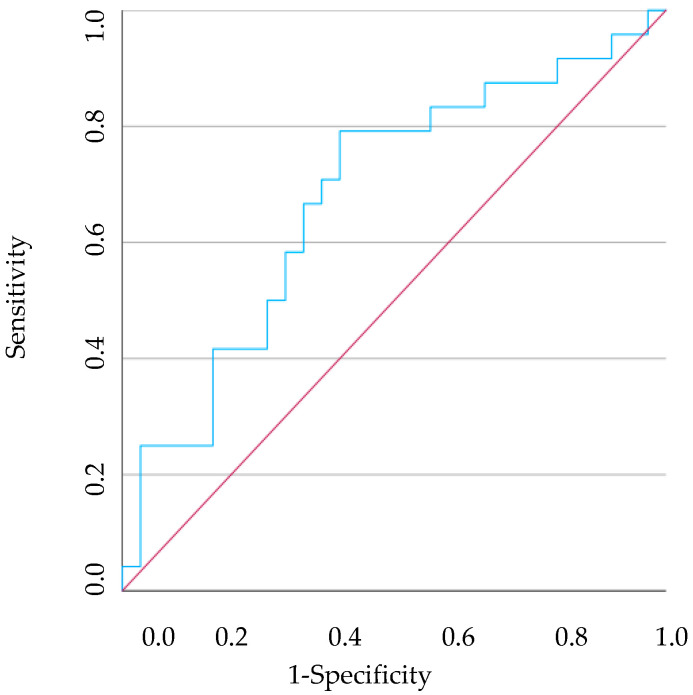
The ROC curve analysis of predicting the PLR between healthy dogs and dogs with periodontitis. The red line illustrates what a random classification would look like, and the blue line represents the model under study.

The ROC curve indicated that PLR could be used to predict the presence of an individual with gingivitis and another with periodontitis with a cut-off point of <81.492 (AUC = 0.691, 95% CI [0.540–0.842], *p* = 0.021, sensitivity 79.2%, specificity 61.5%) (Figure 8) and <82.118 (AUC = 0.679, 95% CI [0.533–0.825], *p* = 0.025, sensitivity 79.2%, specificity 60.0%) (Figure 9), respectively.

## 4. Discussion

The present study evaluated certain HRs in different stages of canine PD. A previous study examined the NLR, PLR, and MPV/PLT in dogs with periodontitis in comparison to healthy dogs and dogs with oropharyngeal tumours [16]. However, to date, no other study has been carried out on HRs in different stages of canine PD, much less in a specific canine breed. It should be emphasised that in this study, none of the dogs in the sample showed clinical signs or laboratory alterations that could be compatible with any other concomitant inflammatory, immune-mediated, or neoplastic pathology, which could influence the HR being analysed.

The HR refers to a ratio that shows the relationship between different types of cells. In our study, we aimed to demonstrate how these indices can vary depending on the degree of canine PD. Only two of the HRs assessed in this study showed no statistically significant differences between the three groups in the analysis.

One factor to consider when analysing our results, which likely influenced all the indices to varying degrees, is the possibility of periodic bacteraemia in dogs with PD [14,22]. Therefore, the more exacerbated periods will influence the HRs analysed here, since there is an activation of both the innate immune response and the acquired immune response, as a result of the absorption of toxins and other bacterial products into the bloodstream [14].

The initial purpose was to determine whether gender and age could influence the HRs analysed. It was concluded that there were no statistically significant differences between males and females in terms of the elements analysed in any of the three groups. In turn, age and HR did not show a statistically significant correlation in any of the study groups, with the exception of MLR in the P group. To date, no studies have analysed in detail the possible influence of gender and age on these HRs. However, in our specific case and in general, there seems to be no influence of gender and age on these parameters. The fact that the MLR in group P showed a statistically significant correlation with age, although this is considered moderate (Spearman’s rho = −0.466), should be investigated in future studies. It has been described that the population of lymphocytes decreases as dogs age [57]. Furthermore, periodontitis, as the last stage of PD, is more prevalent in older dogs [4]. These facts would lead us to think that MLR would increase with age, which was not the case here. Therefore, more studies are needed to understand the possible influence of age on HR.

The first haematological ratio analysed, the NLR, reflects the ratio between neutrophils and lymphocytes, and is considered a biomarker of systematic inflammation, even showing correlations with inflammatory markers such as C-reactive protein [58,59]. Moreover, it reflects two complementing immune pathways [59,60]: lymphocytes carry out adaptive immunological responses, while neutrophils produce non-specific inflammation [59,61] and are cellular elements involved in the body’s first line of defence against infections [62]. A high number of neutrophils would be expected in dogs with periodontitis, as has been observed in humans [59,63,64]. However, the NLR in P group was the lowest, and there were even statistically significant differences between C group versus P group and G group versus P group. Furthermore, it is important to note the decrease in this haematological ratio between clinically healthy dogs and dogs with periodontitis, is, the natural progression of the disease. Given the similarities between the dog, as animal model for PD [12], and humans, it is logical to extrapolate to our case. Thus, this decrease in NLR may be due to an increase in lymphocytes. During periodontitis, there is a chronic inflammatory phase in which the adaptive immune response also becomes relevant [65,66,67], mainly due to lymphocytes [68]. Inflammation induced by persistent microbial harm is what induces T lymphocytes to differentiate [69,70]. Through cytokine release and osteoclast formation regulation, activating distinct T lymphocyte subtypes regulates chronic inflammation. It is a major factor in deciding whether an inflammatory lesion will result in tissue-destructive periodontitis [70,71]. This specific type of lymphocyte has a major impact on the resorption of alveolar bone and is more prevalent [65,70,72]. Moreover, T lymphocytes are key elements to keep the gingival environment in equilibrium [70,71]. In addition to T lymphocytes, B lymphocytes must also be taken into account. B lymphocytes are essential for gingival homeostasis, due to the production of antibodies against different periodontal pathogens, what contributes to the host protection [71]. This leads us to conclude that as canine PD progresses, the role of lymphocytes is more prominent than that of neutrophils, given the chronic inflammatory stage of periodontitis compared to individuals with gingivitis or clinically healthy individuals. Furthermore, the NLR is a more reliable indicator than analysing neutrophils and lymphocytes individually [59,73], which was also demonstrated in this study, since the only statistically significant difference that existed in terms of cellular elements was between the lymphocytes in the C group and the P group.

In the present study, we identified statistically significant differences in the PLR between healthy individuals and individuals with gingivitis, as well as between healthy individuals and individuals with periodontitis. It can therefore be seen that the PLR value is significantly lower in G group and P group compared to C group. Once again, this may be due to the increase in lymphocytes as a result of the chronic stimulation of the immune system due to the inflammatory process that exists in cases of PD [65,66], which could play a key role in the development of other systemic diseases [22,74]. Note that locally produced chemokines and cytokines in inflammatory periodontal tissues contribute to the recruitment of B and T lymphocytes [14], which can stimulate, inhibit, or even kill microorganisms or infected host cells [66]. Inflammation and periodontal tissue damage may result from this action [66,75,76]. The trend towards a higher number of lymphocytes in cases of periodontitis had already been reported in human patients by Iqbal et al. [77], which is in line with our study. Platelet count and also MPV are markers of platelet activation [16,78], with platelet count elevated in chronic inflammatory states [14,42,79]. There are several studies associating platelet activation with PD [14,80,81], and our results lead us to believe that platelet activation and function are in fact more prominent in canine PD than the platelet count itself, as was described by Lu et al. [59]. In addition, the increase in the number of lymphocytes will be more evident, which consequently means that PLR is lower in the groups with gingivitis and periodontitis.

We did not find significant differences in MPV/PLT between any of the three different groups. For this reason, this haematological ratio cannot be considered as a biomarker of canine PD, since there are no statistically significant differences between the various stages. The absence of statistically significant differences in the three comparisons made between the different groups in relation to MPV/PLT can be explained by the fact that there were no significant haematological changes in the parameters used to calculate these ratios. This is contrary to information published in the literature, in which periodontitis patients tended to have an increased platelet count [14], size, and shape [80]. However, in our study, no distinction was made between the existing grades within the periodontitis stage (grade 2, 3 or 4), in contrast to what Mutthineni et al. determined [80], since a distinction was made between moderate periodontitis and severe periodontitis. This may have influenced the failure to obtain statistically significant differences using this ratio.

The other haematological ratio in which there were no statistically significant differences between the groups was MLR. In other words, it seems that canine PD has no impact on MLR. However, it should be remembered that the MLR was the only haematological ratio that showed a statistically significant correlation with age in the P group. Therefore, age may have had an effect on the MLR results. This ratio includes the values of monocytes and lymphocytes, both of which are considered relevant cells in the pathogenesis of PD [82]. In the specific case of monocytes, they are an important element of the innate immune system [46]. When there is an infection with a consequent inflammatory response, monocytes migrate to the inflamed tissues [46]. They then differentiate into macrophages, which kill pathogens by phagocytosis while at the same time producing inflammatory cytokines and reactive oxygen species [46]. In addition, the production of a wide range of pro-inflammatory cytokines also occurs when T lymphocytes are activated [82]. These cytokines cause a succession of events that eventually lead to the progressive loss of the alveolar bone, which supports teeth [71,82]. In the specific case of interleukin-1, this cytokine leads to an exacerbation of the inflammatory response [83,84,85]. It also induces enzymes that degrade the extracellular matrix and, consequently, promote osteoclastic bone resorption [83,84,85]. Although monocyte values were higher in the G group and P group than in the C group, they did not show statistically significant differences compared to the C group, which may have influenced the conclusions of statistical interest in the MLR. This increase in monocytes has been described previously by Buhlin et al. [86] and, in this case, in a statistically significant way. The lack of differences between the groups in terms of MLR suggests the presence of a certain balance between the innate immune response and the adaptive immune response, mediated, among other cells, by monocytes and lymphocytes, respectively. Unfortunately, there are few studies analysing MLR and its importance in canine pathologies [55,87], and our study is the only one to analyse this haematological ratio in canine PD. It would therefore be important to carry out more research into this ratio and it may be interesting, as in the case of MPV/PLT, to substage periodontitis in order to better understand the potential changes in this ratio with the disease.

The lower and statistically significant PNR in the G group compared to the C group may be a direct consequence of the increase in the number of neutrophils. It is widely recognised that the first stage of canine PD is gingivitis [2,5,9]. Later, in the absence of adequate treatment, periodontitis appears, which is considered a chronic inflammatory phase [3]. In cellular terms, neutrophils act as the first line of the innate immune system in the presence of acute infections [14,63], which aligns with this study. As gingivitis is the first stage of canine PD, it is only natural that there should be an increase in the number of neutrophils in group G compared to group C. However, it should be remembered that acute inflammation also leads to the activation of other cells involved in the immunity response, such as platelets [63]. Even so, in this specific case, the increase in neutrophils is still more prevalent. The fact that there were no statistically significant differences in the PNR between clinically healthy individuals and those diagnosed with periodontitis, as well as between the two stages of the disease, once again suggests an adaptive immune response [75], in which T helper lymphocytes contribute to the progression of the disease [67,68]. Thus, acquired immunity becomes more prominent as the disease progresses, causing neutrophils to lose prominence, as cellular elements belonging to the innate immune response.

This study also aimed to analyse the diagnostic accuracy of the HR in predicting the presence of gingivitis or periodontitis, with data obtained through the ROC curve. The NLR in the P group was found to be significantly lower than in the C group. Furthermore, an NLR < 2.577 could differentiate a dog with periodontitis from a clinically healthy dog. On the other hand, the PLR in G and P group was significantly lower than in the C group and a PLR < 81.492 could differentiate a dog with gingivitis from a clinically healthy dog, just as a PLR < 82.118 may identify a dog with periodontitis. Finally, the PNR in the G group was significantly lower than in the C group and could have the ability to discriminate between a dog with gingivitis and a healthy dog when the PNR < 34.490. The only drawback is the sensitivity and specificity obtained, as all from this analysis have poor discriminatory power. This leads us to believe that the exclusive use of these HRs will not be viable. A careful and detailed stomatological-dental assessment, including intraoral radiographs, will lead to a more accurate diagnosis of canine PD. However, it is also important to emphasise that in a veterinary appointment in which a blood count is performed, more specifically in dogs in which it is difficult to assess the oral cavity without sedation and in which there are no other diseases detectable, the use of these HRs could be an added value. A routine blood test will allow us to demonstrate the systemic impact caused by canine PD. Importantly, this fact will facilitate communication between the veterinarian and the owner, empowering them with knowledge of the importance of early diagnosis and intervention in canine PD, and their role in their pet’s health.

Although it was not the main objective of this study, we also have to mention the CBC’s red line. Although there are some studies that associate chronic stages of PD with a reduction in red blood cell parameters [14,88,89,90], in our case, exactly the opposite happened, since there is a significant and increasing trend in RBC, HGB, and HCT as the disease progresses. However, all parameters of the three groups under study were within the reference range, which is in line with what had already been reported by Aljohani et al. [91]. Therefore, additional studies will be necessary to understand the reason for this increase in RBC, HGB, and HCT in cases of periodontitis.

## 5. Limitations

It is essential to mention the limitations that the authors of this study encountered while preparing it. The main limitation, related to the fact that this is a retrospective study, was the lack of blood smears to evaluate potential platelet aggregates and to confirm differential leukocyte counts. However, the haematology analyser used in this study is suitable for obtaining the analyses in question and is widely used in small animal haematology [92]. In addition, the fact that this was a retrospective study meant that only clinical data that was properly discriminated in terms of the examination of the oral cavity was analysed to be able to fit it into one of the three groups under study. This meant that the number of dogs in the study sample was smaller than desirable.

## 6. Conclusions

To our knowledge, this is the first study to analyse five different HRs in canine PD in a single dog breed, in this case the Portuguese Podengo.

In veterinary medicine, the CBC is performed frequently and is considered a minimally invasive and relatively inexpensive complementary means of diagnosis. The HRs that are generated can be simply calculated and without additional cost. Consequently, these HRs could be useful as biomarkers for diagnosis and, eventually, for monitoring a given disease. Our study was able to identify statistically significant differences in three HRs in different stages of canine PD. The NLR was significantly lower in dogs with periodontitis compared to healthy dogs and dogs with gingivitis. Compared to dogs with gingivitis or periodontitis, clinically healthy canines had a much greater PLR. Finally, the value of the PNR showed noticeable differences between dogs who were healthy and dogs who had gingivitis.

Although there are significant differences between the groups in three of the HRs analysed, the results obtained in the present study demonstrate that the exclusive use of NLR, PLR, and PNR is not discriminatory enough to achieve an accurate diagnosis of gingivitis or periodontitis and should always be complemented with a detailed stomatological-dental examination. Nevertheless, given the results obtained, we can say that NLR, PLR, and PNR could be useful as inflammatory biomarkers in canine PD.

Due to the scarcity of studies on HRs in canine PD, we believe that this is an area that will be increasingly explored. Choosing a specific breed is an advantage, and studies carried out on other breeds should be implemented since canine PD has a strong genetic component. As has become a trend, there is an increasing use of specific breeds to determine specific reference intervals, which leads us to believe that this study could be the beginning of this. Therefore, by focusing on developing specific reference intervals for each breed, it will be possible to adopt a more precise and detailed approach to each animal. This trend can be seen in other areas of veterinary medicine, such as cardiology and small animal imaging, and there are already established parameters for certain breeds. In the future, it would be interesting to carry out one or more multi-centre studies with a much higher number of Portuguese Podengo dogs to determine the reference intervals for HRs in healthy dogs, which will facilitate comparative analysis with dogs with a certain pathology. Furthermore, it will also be interesting to determine these HRs in the substages of periodontitis and understand their evolution in dogs undergoing periodontal treatment. 

## Figures and Tables

**Table 1:** General characterisation of dog group sample (*n* = 80).

Group	Median (Range) Age of Dogs (Years)	Median (Range) Weight of Dogs (kg)	Number of Female Dogs (%)	Number of Male Dogs (%)
C	2 (1–8)	13.0 (10.0–16.0)	14 (58.3%)	10 (41.7%)
G	2.5 (1–9)	13.0 (9.0–17.0)	14 (53.8%)	12 (46.2%)
P	3 (1–11)	13.0 (9.0–17.0)	17 (56.7%)	13 (43.3%)

Abbreviations: C: control group; G: gingivitis group; P: periodontitis group.

**Table 2:** CBC parameters in C, G, and P groups and respective significance.

Variable	Unit and Reference Range	Control Group	Gingivitis Group	Periodontitis Group
WBC	6.000–17.000 × 10^9^/L	11.300 ± 3.410 a	13.300 ± 3.150 b	12.300 ± 3.920 b
NEU	3.620–12.300 × 10^9^/L	7.560 ± 2.500 b	8.880 ± 2.690 b	7.770 ± 2.500 b
LYM	0.830–4.910 × 10^9^/L	2.150 (1.690–2.720) a	2.600 (2.120–3.290) b	2.830 (2.170–3.630) b
MON	0.140–1.970 × 10^9^/L	0.650 (0.520–0.778) b	0.735 (0.542–0.895) b	0.675 (0.505–0.925) b
EOS	0.040–1.620 × 10^9^/L	0.595 (0.347–0.945) b	0.770 (0.578–0.938) b	0.560 (0.330–0.795) b
RBC	5.100–8.500 × 10^12^/L	6.290 ± 1.180 b	6.340 ± 0.822 b	7.010 ± 0.808 a
HGB	110.000–190.000 g/L	151.000 ± 26.500 b	153.000 ± 19.000 b	168.000 ± 17.900 a
HCT	33.000–56.000%	42.600 ± 7.140 b	43.300 ± 5.080 b	48.000 ± 4.820 a
MCV	60.000–76.000 fL	68.100 ± 4.780 b	68.400 ± 3.870 b	68.700 ± 3.480 b
MCH	20.000–27.000 pg	24.100 ± 1.720 b	24.100 ± 1.470 b	24.100 ± 1.450 b
MCHC	300–380 g/L	351 (349–356) b	354 (347–358) b	352 (344–357) b
RDW-CV	0.125–0.172	0.138 (0.132–0.143) b	0.139 (0.133–0.146) b	0.139 (0.134–0.145) b
RDW-SD	33.200–46.300 fL	36.900 (35.300–38.300) b	37.200 (35.900–38.700) b	38.000 (36.400–39.100) b
PLT	117.000–490.000 × 10^9^/L	326.000 (241.000–376.000) b	237.000 (156.000–274.000) b	260.000 (230.000–260.000) b
MPV	8.000–14.100 fL	10.100 (9.470–10.300) b	10.100 (9.430–11.300) b	10.000 (8.930–10.800) b
PDW	12.000–17.500	15.800 (15.500–16.300) b	15.700 (15.400–16.600) b	16.000 (15.700–16.800) b

Abbreviations: WBC: white blood cells; NEU: neutrophil count; LYM: lymphocyte count; MON: monocytes count; EOS: eosinophil count; RBC: red blood cells; HGB: haemoglobin; HCT: hematocrit; MCV: mean corpuscular volume; MCH: mean corpuscular haemoglobin; MCHC: mean corpuscular haemoglobin concentration; RDW-CV: red cell distribution width expressed as coefficient of variation; RDW-SD: red cell distribution width standard deviation; PLT: platelet count; MPV: mean platelet volume; PDW: platelet distributed width. Different letters mean statistically significant difference.

**Table 3:** The statistical results obtained in the HR of the C, G, and P groups in relation to gender and age.

	Gender			Age		
	C Group	G Group	P Group	C Group	G Group	P Group
Variable	*p* Value					
NLR	0.666	0.560	0.113	0.227	0.124	0.444
PLR	0.403	0.527	0.133	0.696	0.073	0.111
MPV/PLT	0.464	0.131	0.477	0.139	0.384	0.453
MLR	0.931	0.494	0.213	0.826	0.335	0.009
PNR	0.796	0.899	0.711	0.170	0.415	0.100

Abbreviations: NLR: neutrophil-to-lymphocyte ratio; PLR: platelet-to-lymphocyte ratio; MPV/PLT: mean platelet volume-to-platelet count ratio; MLR: monocyte-to-lymphocyte ratio; PNR: platelet-to-neutrophil ratio.

**Table 4:** Haematological ratios in C, G, and P groups.

	C Group (Median, 25th and 75th Percentiles)	G Group (Median, 25th and 75th Percentiles)	P Group (Median, 25th and 75th Percentiles)
NLR	3.460 (2.540–4.490)	3.320 (2.420–4.460)	2.430 (1.980–3.220)
PLR	119.000 (85.700–210.000)	76.900 (63.800–117.000)	78.300 (59.700–123.000)
MPV/PLT	0.031 (0.026–0.041)	0.046 (0.034–0.067)	0.040 (0.033–0.051)
MLR	0.331 (0.239–0.379)	0.272 (0.212–0.316)	0.228 (0.186–0.288)
PNR	40.400 (32.000–52.500)	26.800 (20.800–35.300)	31.800 (25.600–45.000)

Abbreviations: C: control group; G: gingivitis group; P: periodontitis group; NLR: neutrophil-to-lymphocyte ratio; PLR: platelet-to-lymphocyte ratio; MPV/PLT: mean platelet volume-to-platelet count ratio; MLR: monocyte-to-lymphocyte ratio; PNR: platelet-to neutrophil-ratio.

## Data Availability

The datasets generated for this study are available under request.
